# Discovery and validation of novel and distinct RNA regulators for ribosomal protein S15 in diverse bacterial phyla

**DOI:** 10.1186/1471-2164-15-657

**Published:** 2014-08-07

**Authors:** Betty L Slinger, Kaila Deiorio-Haggar, Jon S Anthony, Molly M Gilligan, Michelle M Meyer

**Affiliations:** Biology Department, Boston College, Chestnut Hill, MA 02135 USA

## Abstract

**Background:**

Autogenous *cis*-regulators of ribosomal protein synthesis play a critical role in maintaining the stoichiometry of ribosome components. Structured portions within an mRNA transcript typically interact with specific ribosomal proteins to prevent expression of the entire operon, thus balancing levels of ribosomal proteins across transcriptional units. Three distinct RNA structures from different bacterial phyla have demonstrated interactions with S15 to regulate gene expression; however, these RNAs are distributed across a small fraction of bacterial diversity.

**Results:**

We used comparative genomics in combination with analysis of existing transcriptomic data to identify three novel putative RNA structures associated with the S15 coding region in microbial genomes. These structures are completely distinct from those previously published and encompass potential regulatory regions including ribosome-binding sites. To validate the biological relevance of our findings, we demonstrate that an example of the Alphaproteobacterial RNA from *Rhizobium radiobacter* specifically interacts with S15 *in vitro*, and allows *in vivo* regulation of gene expression in an *E. coli* reporter system. In addition, structural probing and nuclease protection assays confirm the predicted secondary structure and indicate nucleotides required for protein interaction.

**Conclusions:**

This work illustrates the importance of integrating comparative genomic and transcriptomic approaches during *de novo* ncRNA identification and reveals a diversity of distinct natural RNA regulators that support analogous biological functions. Furthermore, this work indicates that many additional uncharacterized RNA regulators likely exist within bacterial genomes and that the plasticity of RNA structure allows unique, and likely independently derived, solutions to the same biological problem.

**Electronic supplementary material:**

The online version of this article (doi:10.1186/1471-2164-15-657) contains supplementary material, which is available to authorized users.

## Background

Over the last two decades numerous RNA regulators have been discovered that range in function from directing development in eukaryotes [1], to controlling bacterial virulence [2] and metabolism [3]. These RNA regulators vary considerably in their sizes (from less than 20 to greater than 200 nucleotides), required processing proteins (e.g. Argonaut, Dicer, RNase P), and mechanisms of action (inhibition of transcription or translation; utilization of Watson-Crick base-pairing to recognize the transcript of interest, or the formation of complex tertiary structure to enable specific protein-binding or metabolite-sensing) [4–7]. This extensive variability suggests that different regulatory RNAs have very diverse evolutionary origins.

The independent evolution of RNA regulators is especially apparent in bacteria due to the large number of sequenced genomes, and the breadth of biological diversity these genomes represent. Several metabolic processes are controlled by completely distinct RNA regulatory mechanisms in different bacterial species. These include methionine biosynthesis, which is regulated by at least three completely distinct *S*-adenosyl methionine (SAM)-binding riboswitch architectures [8–11], and glucosamine metabolism where both a glucosamine-6-phosphate responsive ribozyme [12] and a series of small RNAs [13] regulate the same pathway in different bacterial species.

The independent derivation of RNA regulators is not restricted to the control of metabolic pathways. In *E. coli*, the over fifty genes encoding ribosomal proteins are localized to approximately twenty transcriptional units, and the stoichiometry of ribosomal proteins is partially maintained through negative autogenous regulation. In this paradigm, excess levels of a given ribosomal protein that are not rapidly assembled into the ribosome, bind a regulatory RNA structure (often located in the 5′- untranslated region) to prevent further transcription or translation of the ribosomal protein effector and other proteins encoded by the operon [14, 15]. Discovered over 40 years ago [16], autogenous regulation of ribosomal protein biosynthesis has been well-characterized in *E. coli* [15]. While several of these RNA regulators appear to be conserved throughout many bacterial species, in most cases these protein-binding sites are narrowly distributed to a few orders of Gammaproteobacteria [17]. In addition there are also many cases where alternative RNA structures that interact with homologous ribosomal proteins are present in different bacterial phyla [18]. Examples include RNAs that interact with ribosomal proteins S4, L20, and S15 [14, 19–24]. The widespread nature of RNA-based autogenous regulation as a mechanism for the control of ribosomal protein synthesis coupled with the narrow distribution of most RNA regulators, and the existence of alternative RNA structures in a few cases, strongly suggests that many similar such mechanisms remain to be discovered in other bacterial phyla.

**Figure 1:**
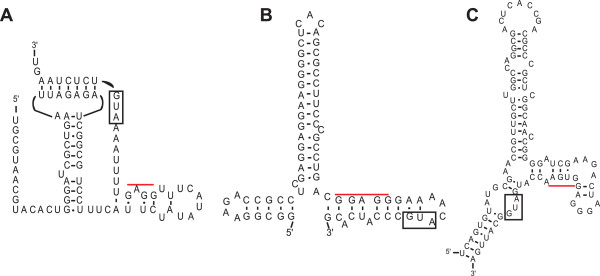
**Previously identified ribosomal protein S15-interacting RNA leader sequences and structures originating from different phyla of bacteria.** Three regulatory RNA structures have been previously reported for ribosomal protein S15. **A**: *Escherichia coli*[22], **B**: *Thermus thermophilus*[24], and **C**: *Geobacillus stearothermophilus*[23]. In each structure the *rpsO* start codon is boxed, and a red bar is placed over the ribosome-binding site.

The RNAs that interact with ribosomal protein S15 are representative of what is likely a common phenomenon. To date, three different RNA structures that interact with ribosomal protein S15 have been identified in *Escherichia coli*, *Thermus thermophilus*, and *Geobacillus stearothermophilus* (formerly *Bacillus stearothermophilus*). Each RNA structure appears to have distinct binding determinants, and they bear little resemblance to the rRNA binding-site for S15 [25, 26]. Yet, each allows negative regulation of *rpsO*, the gene encoding S15. Despite their shared function, the RNA structures show no obvious sequence or structural similarity (Figure 1). While this collection of RNA regulators already highlights RNA structural diversity, examination of their phylogenetic distributions indicates that most bacterial phyla have no previously described S15 regulation [17, 18]. This suggests that there are many more RNAs remaining to be described.

In this work, we implement a framework for computational identification of structured RNA in bacterial genomes and apply it to genomic regions proximal to the S15 coding region (*rpsO*) to assess the diversity of S15-interacting RNAs in bacteria. Our search resulted in many putative structured RNAs across different phyla of bacteria. Sequence alignments corresponding to several of these putative RNA structures were further examined to determine phylogenetic distributions and identify transcription start sites from available RNA-seq data. Additionally, to establish the biological relevance of our results, we experimentally demonstrate that one of these RNAs, originating from the alphaproteobacterium *Rhizobium radiobacter* (also called *Agrobacterium tumefaciens*), has the expected biological function. We validate specific interactions between the predicted RNA structure (Rra-RNA) and the S15 protein from the same species (Rra-S15) using *in vitro* binding assays, and pinpoint regions of the RNA important for protein-interaction using mutagenesis and truncation. The RNA’s secondary structure is further confirmed using structural probing assays. Finally, we also demonstrate that the Rra-RNA regulates gene expression in response to Rra-S15 using an *E. coli* surrogate reporter system.

## Results and discussion

### Comparative genomics identifies several putative RNA structures associated with *rpsO*

**Figure 2:**
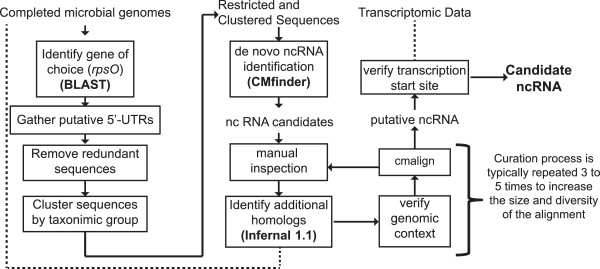
**Overview of comparative genomic pipeline: Genomic Analysis for Illuminating Structured RNA (GAISR).** Putative 5′-untranslated regions (5′-UTRs) of our gene of interest (*rpsO*) are identified within completed microbial genomes and clustered by their taxonomic group. CMfinder is used to identify potential conserved RNA structures within these sequence clusters [30]. Following RNA structure identification, the candidate RNA structures are manually inspected and additional homologs are identified using Infernal 1.1 [31]. The genomic context of putative homologs is assessed, and they are incorporated into the alignment using cmalign. The alignment is then typically manually inspected to identify potential pseudoknots or other regulatory features and the curation process may be repeated several times. Finally, transcriptomic data is sought to identify the transcription start site of the putative ncRNA.

To identify putative RNA structures associated with the coding region for ribosomal protein S15 (*rpsO*), we implemented a computational pipeline, GAISR (Genomic Analysis for Illuminating Structured RNA, Figure 2) for *de novo* ncRNA discovery and candidate refinement. GAISR is based on existing RNA discovery pipelines [27] that have been very successful at identification of ncRNA candidates [28, 29]. GAISR utilizes several pre-existing tools, including CMfinder, a *de novo* ncRNA discovery tool [30], and Infernal 1.1, an RNA homology search tool [31] to streamline sequence selection, identify potential ncRNAs, and efficiently detect additional homologues for putative RNA structures. We used GAISR to examine the genomic region corresponding to the 5′-untranslated region of the gene encoding S15, *rpsO,* in fully sequenced bacterial genomes. From the initial search we identified 52 potential ncRNA sequences, originating from 16 initial phylogenetic sequence clusters.

From these initial sequences, we identified five promising RNA structures. Among these structures were the two known RNAs that allow regulation of *rpsO* in Firmicutes and Gammaproteobacteria [17, 18]. Of note, the RNA structure reported for *Thermus thermophilus* was not identified by our search, suggesting that more RNAs may be present that were not uncovered here. There are several potential reasons for this result including biases in sequence coverage (there were only 19 sequences derived from Deinococcus/Thermus available for analysis), and our use of a single RNA discovery tool for identification of RNA structures may limit our ability to identify putative RNA structures. No tool for RNA *de novo* discovery is designed to identify potential pseudoknotted structures, yet these are very common in biologically functional RNAs [32]. The pseudoknotted structures we have identified (e.g. from Gammaproteobacteria) are typically identified as individual helices by CMfinder and manually merged during the curation process.

**Figure 3:**
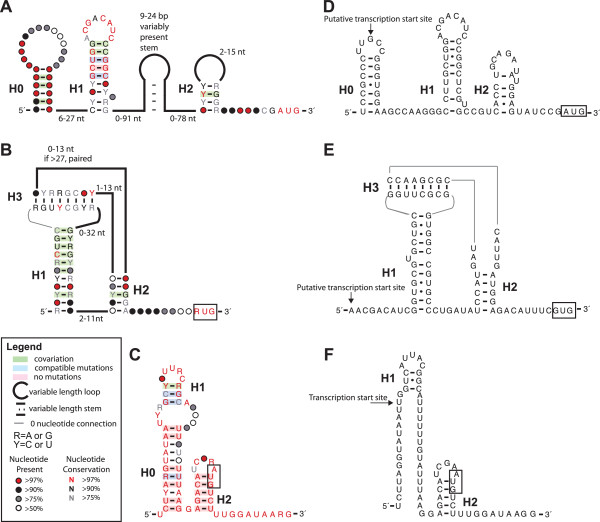
**Consensus diagrams of novel putative RNA structures and individual examples used for transcriptomic analysis.** Novel regulatory RNAs we have identified upstream of the *rpsO* operon. H0 helices were originally predicted by comparative genomics, but not supported by transcriptomic analysis and therefore are unlikely to be biologically relevant. **A**: RNA originating from Alphaproteobacteria, **B**: RNA originating from Actinobacteria, and **C**: RNA originating from Chlamydia. **D**: Alphaproteobacterial RNA example originating from *Rhodobacter spaeroides* (NC_011963.1) showing putative transcription start site determined from analysis of RNA-seq reads (Additional file 4: Figure S1A) [34]. **E**: Actinobacterial RNA example originating from *Mycobacterium tuberculosis* (NC_000962.3) showing putative transcription start site determined from analysis of RNA-seq reads (Additional file 4: Figure S1B). **F**: Chlamydia RNA example originating from *Chlamydia trachomatis* with previously determined transcription start site (NC_010280.1/275170) [37].

Alignments corresponding to the three promising novel structures were curated and additional examples identified using Infernal homology searches. In addition, the phylogenetic distribution of each putative ncRNA was examined, and each alignment was compared with existing RNA-seq data to identify regions likely to be within the *rpsO* transcript. Consensus diagrams of the three candidate ncRNAs are shown in Figure 3 (A-C) and the alignments that correspond to these structures may be found as Additional files 1, 2 and 3. RNA-secondary structures determined from analysis of large phylogenies are often well defined by co-varying nucleotide positions. However, individual sequences corresponding to the RNA structures we identified contain extensive variability including many non-canonical base-pairs and variable-length regions outside of the very well-conserved regions that are likely directly involved in protein-binding. Thus the secondary structure predictions in Figure 3 should be considered tentative. However, the degree of conservation observed here is consistent with that observed for other ribosomal protein-interacting regulatory RNAs that have been experimentally validated in the past [17, 18]. Therefore despite the sequence and structure variability, we believe that the RNAs we identified are likely to have a regulatory function.

### RNAs identified are diverse in sequence and secondary structure

Our first RNA (Figure 3A) was identified in greater than 90% of species within the Alphaproteobacteria orders of Rhizobiales, Rhodobacterales, Rhodospirillales, Caulobacterales, and Sphingomonadales. However, only a single example of the RNA was found in a Rickettsiales species (from 58 genomes explored), potentially reflective of genome reduction in most Rickettsiales species [33]. Our original putative RNA structure included three predicted pairing elements (H0-H2). In ~50% of examples there is also a long-linker region between H1 and H2 (up to 400 nt) that is typically base-paired, although the precise position of this base-pairing within the sequence does not appear to be well-conserved (see Additional file 1 for alignment). The most highly conserved portion of the putative RNA is the H1 helix. This helix shows extensive evidence of co-variation and the loop region is highly conserved suggesting that it is important for protein binding. The H2 helix is less conserved, but typically encompasses a putative ribosome-binding site in the 3′ portion. While H0 shows some co-variation, the loop region is not well conserved in sequence or length. In addition, transcriptomic analysis of RNA-seq data derived from *Rhodobacter spaeroides* [34] (Additional file 4: Figure S1A) suggests that the 5′ portion of this pairing element is not transcribed (Figure 3D), thus we believe that the originally predicted H0 pairing element is likely not part of the biologically relevant RNA.

Our second RNA (Figure 3B) was identified mainly in the Actinomycetales order of Actinobacteria. The putative RNA structure contains a kissing-loop pseudoknotted structure that bears faint resemblance to the RNA structure originating from *E. coli* (Figure 1A)*,* and there are weakly scoring homologs that appear in various Gammaproteobacteria (e.g. Pseudomonas) lacking the known *E. coli* S15 regulator [17]. However, the closing pseudoknot occurs prior to any potential regulatory sequences suggesting that the “entrapment” mechanism proposed for the *E. coli* RNA is not likely to play a role here [35, 36]. Like the RNA described above, a ribosome-binding site is apparent in the 3′ portion of the H2 helix, suggesting a potential translational regulatory mechanism (see Additional file 2 for alignment). Analysis of RNA-seq data from *Mycobacterium tuberculosis* (Additional file 4: Figure S1B), suggests that the transcription start site for this RNA is approximately 10 nucleotides upstream from the start of the first predicted pairing element (Figure 3D).

Our third RNA originates from Chlamydia, and is the one in which we have the least confidence, mainly due to the limited sequence diversity available for analysis (Figure 3E, see Additional file 3 for alignment). However, there is a very strongly conserved hairpin overlapping start codon of *rpsO* in approximately 30 sequenced strains of Chlamydia and a second potential short pairing element displaying some covariation and compatible mutations. In our original prediction, this hairpin was significantly extended (H0). However, pre-existing analysis of transcript start sites in *Chlamydia trachomatis* indicates that the transcript start site is just upstream of H1 (Figure 3F) [37]. Therefore we believe that H0 is likely not part of the biologically relevant RNA. Notably, very few regulatory RNAs have been identified in Chlamydia. Only examples of the TPP and cobalamin riboswitches have been identified in this class of bacteria [38], and in these cases there appear to be only isolated sequences rather than elements that are conserved in many genomes.

The process of curating our original alignments, and in particular the incorporation of RNA-seq data, was critical for narrowing our focus to the portions of the predicted RNAs that are most likely to be biologically relevant. In two cases, transcriptomic data allowed us to determine that putative hairpins predicted through comparative genomics are unlikely to be part of the transcript. Our analysis exemplifies that in assessing the biological relevance of a given ncRNA candidate it is important to determine whether a putative RNA is actually transcribed as well as identify the transcription start site of the RNA candidate [39]. Thus, archives that consolidate RNA-seq data, and provide easily accessible read-depth information for many bacterial species are of great importance moving forward in RNA comparative genomics.

### RNA from alphaproteobacterium *Rhizobium radiobacter*specifically interacts with S15 protein

**Figure 4:**
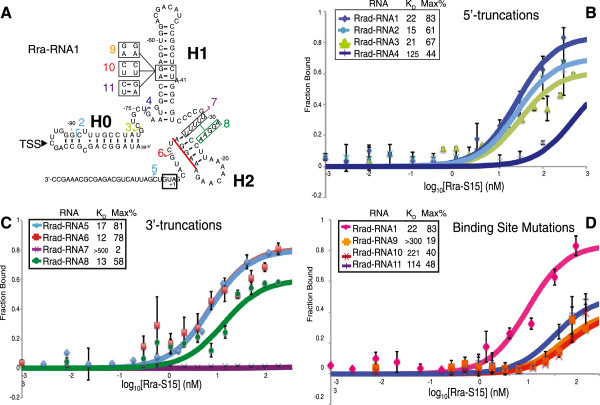
***In vitro*** **nitrocellulose filter-binding assays confirm the transcription start site as well as indicate H1 as the region essential for Rra-S15 binding. A**: Truncation sites and specific mutations to the Rra-RNA. The start codon AUG is boxed, and a red bar is over the ribosome-binding site. **B**: 5′ truncations; **C**: 3′ truncations; **D**: Putative binding-site mutations. Each curve represents at least three independent replicates. For the purposes of comparison, the data from Rra-RNA1 was repeated on graphs **B** &**D**. Reported K_D_ measurements represent the protein concentration at which half of the maximum percentage of Rra-RNA is protein bound. Max% refers to the maximum percentage of Rra-RNA that interacts with Rra-S15 in this *in vitro* assay (see Methods for calculations).

To experimentally validate the biological relevance of our results, we further examined an example of the Alphaproteobacterial RNA originating from *Rhizobium radiobacter* (NC_003062, organism also known as *Agrobacterium fabrum* strain C58, and formerly known as *Agrobacterium tumefaciens* strain C58). The sequence from *R. radiobacter* conforms well to our consensus structure, containing the highly conserved H1, and the predicted H2 pairing element. In addition, this sequence is one where the region directly preceding our transcription start site has the potential to base-pair with the 5′-most portion of the RNA. We designated this helix H0 due to its position 5’ of the predicted transcription start site. We first tested the full-length version of the RNA (nucleotides -108 to +27) and called it Rra-RNA1 because it was the first RNA tested from this organism (Figure 4A). To examine whether this RNA interacts specifically with S15 protein from the same organism (Rra-S15) we utilized filter-binding assays [40]. These assays confirmed that Rra-RNA1 binds Rra-S15 with nanomolar affinity (Figure 4A,B; K_D_ = 22.2 ± 0.7 nM). This value is similar to those reported for the interactions between S15 and the RNA structures originating from *E. coli* (45 nM) [35], *G. stearothermophilus* (20 nM) [23], and the *T. thermophilus* (5 nM) [24].

### Truncation analysis suggests that Rra-S15 minimal binding site includes both H1 and H2

To experimentally investigate the validity of the putative transcription start site we constructed several 5′ truncations to the Rra-RNA1 sequence and tested their ability to bind Rra-S15. Based on the putative transcription start site derived from analysis of RNA-seq data from *Rhizobium spaeroides* (at C-95 according to our alignment), the potential H0 helix predicted from comparative genomics in the absence of RNA-seq analysis (Figure 3A,D) is unlikely to be necessary for Rra-S15 binding. We performed 5′-RACE for this RNA to further identify the transcription start site. Although C-95 was one of the 5′-ends identified (Additional file 4: Figure S2), this experiment provided multiple 5′-ends and was ultimately inconclusive. Truncations Rra-RNA2 (nucleotides -91 to +10) and Rra-RNA3 (nucleotides -79 to +10) appear to have negligible effects on Rra-S15 binding (K_D_ =14.5 ± 6.1 nM and 21 ± 4.8 nM, respectively) (Figure 4A,B). These results indicate that all bases upstream of nucleotide -79 are not required for binding Rra-S15, consistent with the putative transcription start site prior to this nucleotide at C-95. Binding was not significantly affected until the RNA was truncated to G-72, Rra-RNA4 (K_D_ =125 ± 106.5 nM) (Figure 4A,B). Collectively, these results suggest the entire H0 stem and loop are dispensable and the C-95 identified during analysis of RNA-seq data from *R. sphaeroides* likely represents the transcription start site.

To identify the minimal protein binding-site, we examined 3′-truncations to the Rra-RNA (Figure 4A,C). In the gammaproteobacterial RNA, the initial amino acid encoding nucleotides of *rpsO* form an integral part of the RNA structure and function [36]. However, removing the coding region of the alphaproteobacterial RNA (Rra-RNA5, nucleotides -108 to +5) has minimal effect on the binding affinity (K_D_ =16.6 ± 10.8 nM). Rra-RNA6 (nucleotides -108 to -6) was designed to remove all bases downstream of the predicted H2; again, this RNA binds Rra-S15 with an affinity better than that of Rra-RNA1 (K_D_ = 11.9 ± 1.8 nM). The observed increase in binding affinity is likely due to removal of potential alternative competing structures, thus allowing a tighter interaction between the protein and the RNA. Rra-RNA7 (nucleotides -108 to -31) was designed to remove all of predicted hairpin H2 including the five uracils (U-26 to U-30) through the putative ribosome binding site (purine-rich sequence from A-8 to A-13), start codon and subsequent protein coding nucleotides. This truncation completely abolishes Rra-S15 binding (K_D_ >500). To assess whether slippage along the predicted H2 might allow the five uracils (U-26 to U-30) to base-pair with the putative ribosome binding site (A-8 to A-13), we mutated the polyuridine to a purine-rich sequence to destabilize this alternative pairing (Rra-RNA8, Figure 4A). This mutant was able to bind Rra-S15 with a similar affinity to the full length Rra-RNA1 (K_D_ =12.5 ± 2.9 nM) suggesting that the pairing we have drawn is one that allows for protein binding. Based on this data we predict the minimal RNA regulatory region includes nucleotides G-79 through U-6, which is fully encompassed by our predicted transcript, and includes both of the predicted pairing elements H1 and H2.

### Mutation analysis suggests a potential binding-site

The most highly conserved portion of this RNA is the H1 stem loop (Figure 3A). Due to its high sequence conservation, we hypothesize that this region is essential for Rra-S15 binding. Mutations made in this stem, Rra-RNA9 and Rra-RNA10, both significantly inhibit binding (Figure 4A,D; K_D_ values of >500 and 221 ± 52.3 nM, respectively). The compensatory mutation, Rra-RNA11, was able to partially recover Rra-S15 binding (K_D_ =114 ± 37 nM). In this compensatory mutant, it is likely an alternative base-pair forms with usually unpaired A-41 and the dynamic equilibrium of the two RNA structures allows, but does not completely restore, Rra-S15 binding. In combination with the truncation experiments above, these results suggest that Rra-S15 binds its RNA regulator in the highly conserved stem-loop structure of H1 but that H2 is still required for binding.

### The *R. radiobacter*RNA allows regulation in response to S15 *in vivo*

To determine whether the Rra-RNA has regulatory activity in addition to S15-binding activity, we conducted *in vivo* reporter assays to assess regulation. To do this we used a GFP reporter to measure expression of the gene following the Rra-RNA in response to different levels of Rra-S15. The RNA sequence was cloned in-frame as a translational fusion with the GFP reporter under the control of the IPTG-inducible *trc* promoter. This construct included the *rpsO* start codon, Shine-Dalgarno sequence, and the first nine codons of the *rpsO* gene to form the RNA-GFP fusion. On a second plasmid, the *R. radiobacter rpsO* coding sequence was placed under the control of an L-arabinose inducible promoter. The pair of plasmids were co-transformed into an *E. coli* K12:Δ*rpsO* strain. We chose to use a surrogate organism, *E. coli*, due to its ease of use and manipulation, and the ability to obtain a knockout organism lacking endogenous S15. Using this GFP *in vivo* reporter system, we assessed the ability of Rra-S15 to regulate gene expression by measuring the GFP levels in the cells in the presence and absence of induced Rra-S15. If the RNA interacts with Rra-S15 to regulate gene expression, we expect to see a decrease in GFP expression in cells expressing Rra-S15 compared to cells not expressing Rra-S15.

**Figure 5:**
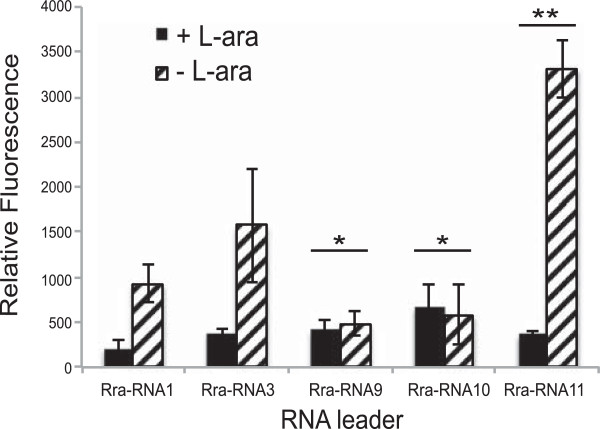
**GFP reporter assays validate Rra-RNA’s regulatory role.** GFP reporter assays validate the regulatory capacity of Rra-RNA in response to Rra-S15. Rra-RNA structure and sequence are the same as described in Figure 4. All relative fluorescence values were calculated by normalizing GFP/OD_600_. All bars are 3+ independent experiments. *indicates p < 0.01; **indicates p < 0.001.

Cells co-transformed with plasmids containing full length Rra-RNA1-GFP, and Rra-S15 were grown in the presence and absence of L-arabinose. The cells grown in the presence of the sugar (induced Rra-S15) displayed a ~4-fold decrease in GFP-reporter expression (Figure 5). Because L-arabinose induces Rra-S15 production, the decrease in GFP reporter expression is likely due to an interaction between the RNA and Rra-S15. Next, to corroborate that our predicted transcription start site at the C-95 allows regulation, the sequence for Rra-RNA3 (nucleotides -78 to +27) was also tested in this system and behaved in a similar manner. These results indicate an RNA sequence starting at the transcription start site derived from *R. spaeroides* is sufficient to allow regulation *in vivo*.

We also examined whether mutations to H1 that abolish Rra-S15 binding would affect regulation. Cells containing either Rra-RNA9-GFP and Rra-RNA10-GFP did not display a significant difference in GFP reporter expression when grown in the presence and absence of L-arabinose. This is likely because both Rra-RNA9 and Rra-RNA10 do not interact specifically with S15 and are unable to regulate the expression of the reporter. However, it should be noted that GFP expression levels in the absence of arabinose were also significantly lower than those observed with the Rra-RNA1 and Rra-RNA3 constructs.

To assess whether the partial compensation of binding observed for RraRNA11 *in vitro* represented a biologically relevant, functional compensation, Rra-RNA11 was also examined in this system. In this case, cells grown without L-arabinose displayed an increased GFP expression level compared to Rra-RNA1, and cells grown in the presence of L-arabinose had a ~10-fold decrease in relative GFP fluorescence (Figure 5). However, the increase in fold-change is solely due to increased RNA11-GFP expression levels and the repressed level of gene expression is comparable between the two RNA elements. Thus, the Rra-RNA11 compensatory mutation that partially restored the *in vitro* RNA-protein interaction also restored the regulatory interaction between the Rra-RNA and Rra-S15. The partial restoration of *in vitro* binding by the compensatory mutant Rra-RNA11 is likely due to the presence of several competing structures formed by the RNA under these conditions. However, the *in vivo* conditions enable the RNA to adopt a secondary structure that increases overall reporter expression and enables regulation in response to S15. Together, these assays indicate that not only the does this RNA interact with Rra-S15 *in vitro*, but it is a biologically relevant regulatory element responding to S15.

### Structural probing confirms predicted secondary structure

To further examine the secondary structure of Rra-RNA in the absence of protein we used several structural probing methods in combination with a minimal RNA construct (Rra-RNA6) including nuclease cleavage assays with (RNase VI and RNase A), and in-line probing. RNase VI cleaves double stranded RNA non-specifically, RNase A cleaves single stranded C’s and U’s, and in-line probing the RNA structure reveals the flexible regions of the RNA structure (and likely single-stranded regions) that are more prone to spontaneous self-cleavage.

**Figure 6:**
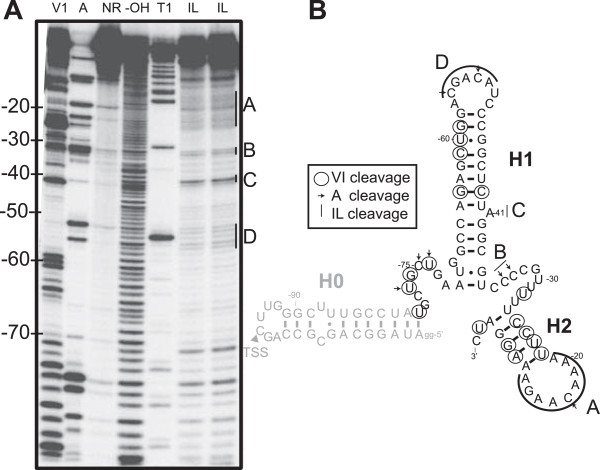
**Structural probing confirms predicted secondary structure. A**: RNase V1 (V1), RNase A (A), no reaction (NR), hydroxyl cleavage (^-^OH), denaturing RNAse T1 (T1), and two independent replicates of in-line probing reactions (IL) where the cleavage products have been separated by denaturing 10% PAGE. Cleaved cytosine and uridine residues in the RNase A reaction and cleaved guanosines in the denaturing T1 reaction were used to map cleavage to the RNA structure, and regions of strong in-line cleavage are labeled. **B**: Mapping of prominent cleavage sites to the structure of Rra-RNA6; bases in black are resolved on the gel. TSS indicates predicted transcription start site. Cleavage sites largely confirm structure anticipated from comparative genomics.

Although the putative stem H2 is predicted in our alignment (Additional file 1), there are many sequences that contain short polypyrimidine sequences that are unpaired in our sequence alignment. These sequences may form alternative pairings with the ribosome-binding site (AG rich region ~8 nucleotides before the translation start site). Based on sequence data alone it is difficult to distinguish which bases are interacting with the ribosome-binding site. However, several lines of evidence indicate that we have identified the correct *in vitro* base-pairing conformation for our putative H2 in the *R. radiobacter* example of the RNA (Figure 6). First, our mutagenesis and truncation analyses indicate that mutating the polyuridine (U-26 to U-30 in Rra-RNA8) does not alter protein-binding activity. This suggests that this region is unlikely to interact with the putative ribosome-binding site (-13 to -8). However, deleting this region and the following hairpin (Rrad-RNA7) abolishes protein binding indicating that H2 is important for protein binding. Second, RNAse V1 cleavage occurs symmetrically in regions that are base-paired in our figure (-8 through -12 and -23 to -28), and RNaseA cleavage occurs at C-17 as would be expected for a loop region. In addition, in-line probing shows that the entire 3′ portion of the molecule is somewhat flexible, from bases -11 through -22 (region A). In conjunction with our mutagenesis results, this strongly suggests the correct pairing-element has been identified.

Consistent with our mutagenesis results, the highly conserved stem H1 (bases U-42 to A-65) is almost certainly double-stranded with a loop from C-49 to A-56. Bases U-42 through C-49 are shielded from in-line attack and there are strong cleavage bands in RNAse VI probing for bases C-43, bases -59 to -61, and G-64. Also, there are no RNAse A cleavage products for any of these uracils or cytosines, suggesting that these nucleotides are not single-stranded. There is also evidence for the predicted loop region in H1. Probing with RNAse A results in cleavage products for C-53 and C-56 and in-line probing reveals that C-53 through A-57 (region D) are flexible. At the base of the H2 stem, we predict a bulged adenosine (A-41) and the highlighted region C of our in-line probing gel corresponds to this bulged base. This data corroborates our other evidence that the region essential for Rra-S15 binding in H1 forms a double-helix.

The nature of the junction between the two predicted helices is still unresolved. This region is not well-conserved so there is little phylogenetic evidence of structure, and in several cases the different assays give conflicting results, which may be the result of multiple folding conformations. The string of uridines from U–27 to U-30 does not appear to be flexible based on in-line probing, is cleaved by RNAase V1, and is not cleaved by RNase A, indicating that the region is not single-stranded. However, there are also no clear binding partners for these nucleotides suggesting that they may be forming a constrained tertiary structure. The string of cytosines that follows this region, C-32 to C-35, do show strong RNase A cleavage suggesting they are single-stranded, and this is corroborated by the in-line cleavage at these positions (region B). However, these bases also display RNAse V1 cleavage indicating that they may sometimes adopt a double-stranded conformation. Nucleotides from -35 to -40 are not cleaved by either RNase V1 or RNAse A, and appear to be structurally constrained. This suggests that they are not necessarily double-stranded, but may be participating in some tertiary structure. Nucleotides -75 to -80, which potentially could interact with these bases, also show conflicting results, cleaving with both RNase V1 and RNase A. We have included the possible base pairing of the nucleotides at the base of H1 in our structure figures (Figures 4 and 6), but these interactions are likely weak. The accumulation of these data strongly suggests that both the predicted pairing elements do form, but the nature of the junction between these two elements remains unclear.

## Conclusions

This work demonstrates the premise that nature may invent many unique ways to solve a single biological problem. In the context of other forms of RNA-based regulation the diversity of distinct RNA structures allowing *cis*-regulation of the *rpsO* operon is nearly unmatched. The only similar example of such diversity in RNA regulators for a specific function are the SAM-binding riboswitches, where more than three completely distinct classes [8–10], and several additional sub-classes with re-arranged or modified secondary structure elements have been characterized [41, 42]. The S15 auto-regulatory RNA structures we identified are quite diverse from one another, and from the existing known characterized S15 regulatory RNAs that originate from *E. coli*, *G. stearopthermophilus*, and *T. thermophilus* [22–24].

All of the previously characterized RNA structures encompass a predicted Shine-Dalgarno sequence, but beyond such regulatory features the RNAs appear to share very few common sequence features or patterns in secondary structure. While the S15-interacting RNAs potentially share some tertiary structure similarities that are not captured in the secondary structure diagrams, previous studies indicate that the *E. coli* S15 does not interact with the regulatory RNA originating from *G. stearothermophilus* [26]. This finding suggests that there may be no single conserved tertiary structure shared by the S15-binding mRNA structures. In the absence of structural data, it remains to be seen whether the structural diversity apparent in natural S15-interacting mRNA structures is a result of RNA’s inherent ability to generate a similar tertiary structure from diverse arrangements of primary and secondary structure [21, 43], or from differences between the S15 protein homologs that lead to distinct pools of potential RNA ligands. From the structures we describe here, it is clear that there are many ways to solve this particular biological problem. An exciting question that stems from this work is precisely how prevalent are RNA solutions within sequence space that allow autogenous regulation in response to S15. Based on the natural diversity of S15-interacting RNAs, we expect that this number is large, and that as more genomes are sequenced and the sensitivity of computational searches increases, additional structures with this function will be identified.

## Methods

### Computational identification of putative RNAs and curation of RNA alignments

*RpsO* was identified in the genomes of fully sequenced bacteria (refseq58-microbial [44]) using tBLASTn [45]. Sequences corresponding to the putative 5′ non-coding regions (500 nucleotides 5′ of the translation start, or the end of the previous gene) in addition to 25 nucleotides of the *rpsO* coding region were collected. Sequences containing >90% sequence identity over >70% of the sequence length were removed as redundant. The remaining sequences were clustered based on taxonomy into groups of 100 or fewer sequences. CMfinder was run on these clusters with the default parameters [30]. The resulting alignments were manually curated to identify the most promising RNA candidates.

Covariance models for each RNA alignment were constructed and calibrated using Infernal 1.1 (cmbuild -F, cmcalibrate –cpu 4), and homologues were identified for each alignment [31]. Cmsearch was performed against a custom sequence database described above using a lenient e-value cut-off of 1.0 (cmsearch –E 1.0 –mid –cpu 4). Sequences were then aligned using cmalign (--mapali –cpu 3 --noprob). Alignments were subsequently manually adjusted as necessary when sequences with variable-length helices and/or loops were added. The search process was repeated approximately 3-4 times per multiple sequence alignment, to expand sequence diversity. During the course of these searches, the alignments were extended at the 5′ and 3′ ends to encompass any potential flanking sequence and pseudoknotted or alternative structures were identified through curation of the alignment. Transcription start sites were identified through examination of mapped read-depths derived from RNA-seq data [34, 46] compiled at AREBA (An RNA Encyclopaedia for Bacteria and Archaea (https://github.com/UCanCompBio/AREBA), or from previously assessed transcription start sites in the literature [37]. Consensus secondary structure diagrams were created from the alignments using GSC-weighting in R2R [47].

### RNA preparation

DNA corresponding to the 5′-UTR of the *rpsO* gene with the T7-promoter appended was PCR amplified from *R. radiobacter* genomic DNA. Mutants 8-10 were generated through QuickChange mutagenesis on Rra-RNA1 template, then PCR amplified using Rra-RNA1 primer set. T7 RNA Polymerase [48] was used to transcribe RNA, and RNAs were purified by denaturing PAGE (6%), bands visualized using UV shadow, and RNA eluted from excised bands in 300 mM NaCl, 1 mM EDTA. Purified RNA was 5′-labeled with ^32^P-ATP [49] and again purified as described above.

### Protein preparation

The *R. radiobacter rpsO* ORF was cloned into pET-HT overexpression vector [50] and transformed into BL-21(DE3) cells (Invitrogen). Protein was over-expressed and cells lysed by sonication using S15 Resuspension Buffer (100 mM Tris/HCl, pH 8.0, 800 mM NaCl, 150 mM MgCl_2_). S15 was soluble and was purified at 4**°**C using non-denaturing FPLC cation exchange chromatography, pH 5.5, with a linear salt gradient (100 mM-1 M NaCl)[51]. A second purification was performed under conditions previously described [52] using pH 8.0 and a linear salt gradient (20 mM – 1 M KCl) at 4**°**C by non-denaturing FPLC cation exchange chromatography. RNAse-free protein fractions were concentrated, analyzed via SDS-PAGE, and buffer exchanged for the S15 Storage Buffer (50 mM Tris/Acetate, pH 7.5, 20 mM Mg-Acetate, 270 mM KCl), Final protein concentration was determined by Bradford assay and stored at 4**°**C.

### Filter-binding assays

RNA binding capability was examined by filter-binding assay (FBA). A fixed amount of 5′-labeled RNA (1000 cpm, <1 nM) was renatured for 15 minutes at 42**°**C, then incubated with serial dilutions of S15 in Buffer A (50 mM-Tris/Acetate, pH 7.5, 20 mM Mg-Acetate, 270 mM KCl, 5 mM dithiothreitol, 0.02% bovine serum albumin), for 30 minutes at 25**°**C. Nitrocellulose membrane (GE Healthcare) was used to collect RNA-S15 and nylon (GE Healthcare) to collect unbound RNA under suction. Membranes were air-dried for 5 minutes and the fraction bound quantified by imaging membranes on a phosphorimager screen. Radioactivity counts per sample on each membrane were measured using GE Healthcare STORM 820 phosphorimager and ImageQuant. For each sample, the fraction bound (Fb) corresponds to the (counts nitrocellulose)/(counts nitrocellulose + counts nylon). To determine the K_D_ and the maximum fraction bound (Max%), the resulting values were fit to the equation: Fb = (Max%*[S15])/([S15] + K_D_) where [S15] corresponds to the concentration of S15 in the reaction. The residuals were minimized using the Solver function in Microsoft Excel to find both the Max% and the K_D_. K_D_ values given in the text represent the mean of 3 or more independent binding assays ± the standard deviation.

### Structural and nuclease probing assays

The RNA-protein binding reaction described above was used for RNAse probing assays. After incubation, 1 uL RNAse A (1 ug/mL, Ambion) or RNAse VI (1:400 dilution of 0.1 U/uL, Ambion) was added and the reaction incubated for 15 minutes at 25**°**C. The nuclease was inactivated with inactivation/precipitation buffer (Life Sciences) and RNA fragments recovered by ethanol precipitation. Precipitated RNAs were suspended in 10 uL Urea Loading solution (Life Sciences) and incubated for 5 minutes at 95**°**C. Five uL of each reaction was loaded on 10% denaturing Acrylamide/Bis-acrylamide gel. The gel was dried and examined using a GE Healthcare STORM 820 phosphorimager and ImageQuant software. Partial hydroxyl cleavage reactions were generated by incubating RNA in Reaction Buffer (50 mM Na_2_CO_3_ pH 9.0, 1 mM EDTA) at 95**°**C for 7 minutes. Denaturing T1 reaction was conducted according to manufacture′s protocol (Ambion). For in-line probing, 5′-labeled RNA was incubated for 40 hours at 25**°**C in Reaction Buffer (20 mM MgCl_2_, 100 mM KCl, 50 mM tris pH 8.3). The reaction was stopped using Urea loading solution (10 M Urea, 1.5 mM EDTA).

### Plasmid construction

The ptrc-RNA-GFP plasmid was constructed from pLac-thiMwt-tetA-gfpuv plasmid [53] as outlined in detail in Additional file 4. Essentially, the RNA sequence and the first nine codons were placed as a translational fusion with GFP replacing the thiamine responsive riboswitch and existing ribosome-binding site. The pre-existing *lac* promoter was replaced with a *trc* promoter. The protein expression plasmid was constructed by amplifying the DNA fragment encoding the *rpsO* gene containing a SacI restriction site and a ribosome-binding site on the 5′ terminus (5′-caagagctcaggaggttttaaaatgtcgattactgcagagcgcaaag) and XbaI site on the 3′ terminus (5′- caatctagattagcggcgaatgccgagagc) from genomic DNA extracted from *R. radiobacter* (ATCC 23308). The PCR product was digested with SacI and XbaI enzymes and inserted into the pBAD33 expression vector (ATCC 87402) digested with the same enzymes.

### *E. coli*regulatory assays

K12: Δ*rpsO E. coli* cells (CGSC# 7154: strain CK1953, *E. coli* Stock Center Yale University) were co-transformed with an RNA and protein plasmid (made competent using the Z-competent buffer system, Zymo Research). Overnight cultures were grown +/- L-arabinose (15 mM), then diluted the next day to OD_600_ = 0.150 in fresh media (LB + 100 ug/mL AMP + 34 ug/mL CHL +/- 15 mM L-arabinose). At log phase, IPTG (2 mM final) was added to induce GFP expression and cells were grown an additional 5 hours. Cells were collected, washed with PBS, then stored in PBS overnight. GFP expression was measured using a SpectraMax M5 fluorimeter (excitation: 395 nm, emission: 508 nm, Molecular Devices). Fluorescence was calculated by normalizing GFP to cell density (GFP/OD_600_).

### Availability of supporting data

The software described in Figure 2 is available under an MIT open license at https://github.com/jsa-aerial/gaisr. Sequence alignments corresponding to the structured RNAs in Figure 3 are available as Additional file 1, Additional file 2, and Additional file 3. Nucleic acid probing data displayed in Figure 6 has been deposited at http://snrnasm.bio.unc.edu.

## Electronic supplementary material

Additional file 1: **Alignment of Alphaproteobacterial RNA.** (TXT 86 KB)

Additional file 2: **Alignment of Actinobacterial RNA.** (TXT 54 KB)

Additional file 3: **Alignment of Chlamydia RNA.** (TXT 5 KB)

Additional file 4: **Supplementary Figures and Methods.** (PDF 272 KB)
