# Supplementary material for: Discovery and validation of novel and distinct RNA regulators for ribosomal protein S15 in diverse bacterial phyla
Source: BMC Genomics. 2014 Aug 7;15(1):657. doi: 10.1186/1471-2164-15-657 (PMC4137082; doi:10.1186/1471-2164-15-657)
Supplement: Supplementary file 4 — Additional file 4: Supplementary Figures and Methods. (PDF 272 KB) [file 12864_2014_6354_MOESM4_ESM.pdf]

Supplemental Figures and Methods for Slinger et al.

Discovery and validation of novel and distinct RNA regulators for ribosomal protein S15 in diverse bacterial phyla

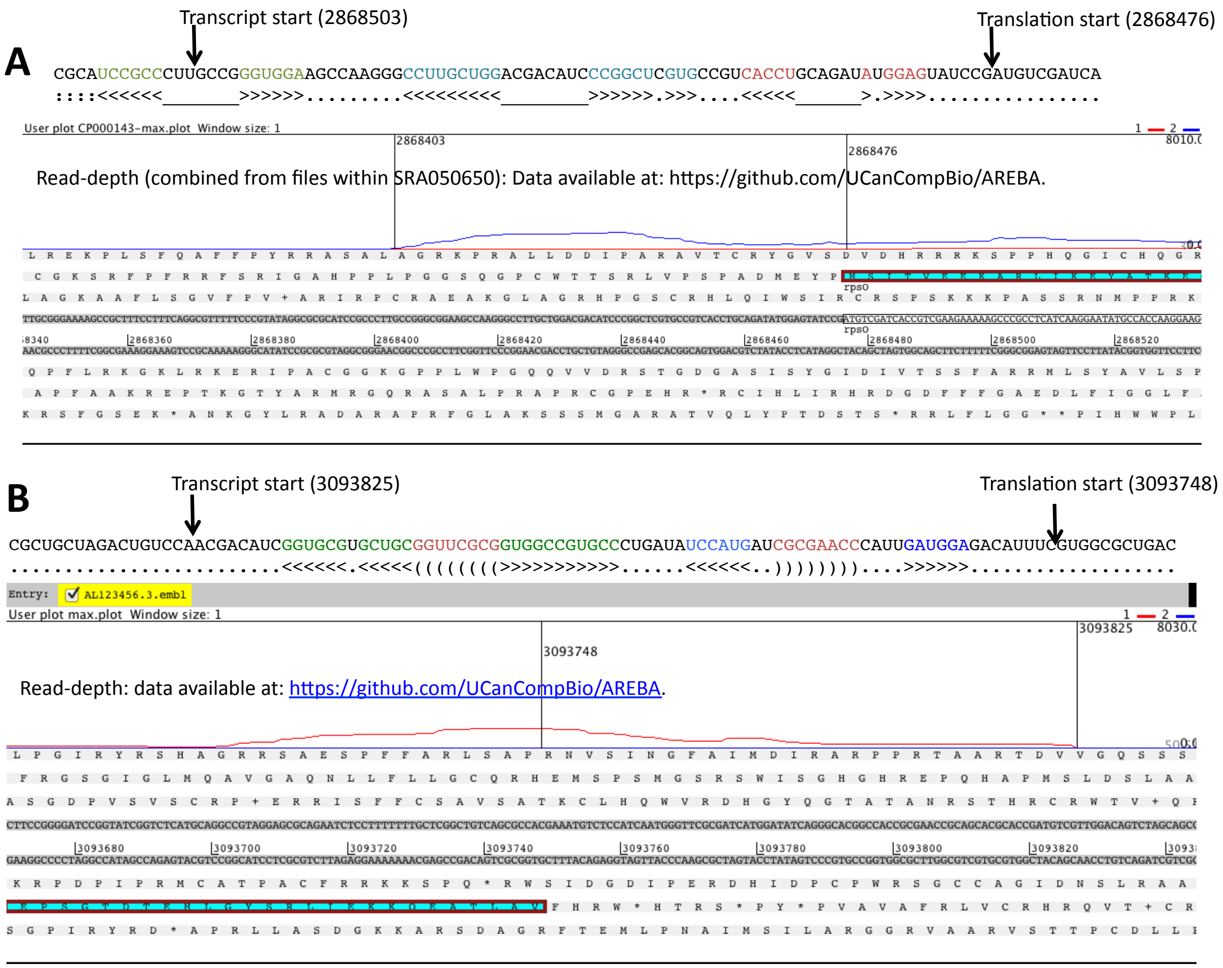

Figure S1: Transcriptomic analysis of Alphaproteobacterial and Actinobacterial RNAs. To determine transcription start sites, we examined both the primary literature and the AREBA archive (A RNA Encyclopaedia for Bacteria and Archaea; Bacterial and Archaeal Transcriptome Meta-analysis Project, https://github.com/UCanCompBio/AREBA) to identify RNA-seq datasets for organisms that contain examples of the putative RNA. In this figure we display read depth plots from the AREBA archive (mapped RNA-seq data) from A: *Rhodobacter sphaeroides* [1] and B: *Mycobacterium tubercuolosis* [2] displayed by the Artemis Genome Browser. This is paired in each case with the predicted RNA sequence and secondary structure annotation. In the case of *R. sphaeroides*, the transcription start site appears within the initially predicted 5'-most pairing element, calling into question whether this putative element is part of the RNA.

***R. radiobacter* genomic *rpsO* genomic region**

AGATCGGCGAAGGCGAGTTCAGGCCGAAGCGGGTTTTTGGCTAACCATACGGCGCGGAGGCGCTGTCAAT
CGCCTCTTTCAATTGCACCCGGAATAGTTTATAGGCAGCGCCAGCTTGGGCTTTGCCTATGCTGCTGAAT
GGCCAGAGCTGGACGACATCCCGGCTCTAGGCGTCCCCGTTTTTCCTTAAAACAAGAAAGGATCGTACGA
TGTCGATTACTGCAGAGCGCAAAGCCGCCCTCATCACGGAATATGCCACCAAGGCAGGCGACACCGGTTC
TCCGGAAGTTCAGGTCGCAATCCTGACCGAGCGGATCAACAACCTGACCGGTCACTTCAAGGACCACAAG
AAGGACAACCACTCCCGTCGTGGCCTTCTGACGCTCGTTTCGAGCCGCCGTTCGCTTCTCGACTATCTGA
AGAAGAAGGACGAAGCCCGTTACACCAAGCTGATCGGTGCTCTCGGCATTCGCCGCTAA

Underlined: Rra-RNA1 full length

Yellow: 1 5’-RACE product

Red: 2 5’-RACE products

Pink: 4 5’-RACE products

Green: protein coding region

Predicted transcription start site based on *R. sphaeroides* transcriptome data.

Figure S2: 5’ RACE results for *rpsO* transcript in *Rhizobium radiobacter*.

Supplemental Methods:

R. radiobacter RNA 5’-RACE

Total RNA was extracted from log phase *R. radiobacter* cells grown in LB and 5’RACE performed using Invitrogen GeneRacer kit. Reverse transcription was conducted using a gene-specific primer (623-RradS15M11R: 5’-atcctttcttgttttaaggaaaaacggg), and the product PCR amplified with an oligo linker-specific primer (9-Forward5’RACE: 5’-gactggagcacgaggacactga) and gene-specific primer (647-RradS15M13R: 5’- gacgcctagagccgggatgtcgt). PCR product was cloned using TOPO-cloning kit (Invitrogen) and sequenced (Eton Biosciences) to identify the transcription initiation site of the *rpsO* transcript. The results of this analysis can be found in Supplementary Figure 2.

Construction of ptrc-GFP plasmid

To change the promoter, oligonucleotides encoding the trc IPTG-inducible promoter flanked by Xho I and EcoRI restriction sites at the 5’ and 3’ termini respectively (5’-gagctgttgacaattaatcatccggctcgtataatgtgtggaattgtgagcggataacaatt-3’), and its reverse complement were chemically synthesized (Eurofins MGW Operon), phosphorylated (T4 polynucleotide kinase, NEB), and annealed. The double-stranded DNA was inserted between the XhoI and EcoRI sites of digested pLac-thiMwt-tetA-gfpuv plasmid, replacing the pLac promoter. DNA fragment containing the RNA leader sequence with restriction sites EcoRI and SalI on the 5’ and 3’ ends respectively was PCR amplified from genomic template using gene specific primers. The PCR product was digested with EcoRI and SalI and inserted into pLac-thiMwt-tetA-gfpuv plasmid digested by the same enzymes, replacing the thiMwt riboswitch sequence. A DNA fragment encoding gfpuv replaced the existing gfpuv-tet reporter in this vector.

References:
1. Giannoukos G, Ciulla DM, Huang K, Haas BJ, Izard J, Levin JZ, Livny J, Earl AM, Gevers D, Ward DV, Nusbaum C, Birren BW, Gnirke A: **Efficient and robust RNA-seq process for cultured bacteria and complex community transcriptomes**. *Genome Biol* 2012, **13**:R23.

2. Arnvig KB, Comas I, Thomson NR, Houghton J, Boshoff HI, Croucher NJ, Rose G, Perkins TT, Parkhill J, Dougan G, Young DB: **Sequence-Based Analysis Uncovers an Abundance of Non-Coding RNA in the Total Transcriptome of Mycobacterium tuberculosis**. *PLoS Pathog* 2011, **7**:e1002342.
